# Supplementary material for: Split-HaloTag imaging assay for sophisticated microscopy of protein–protein interactions in planta
Source: Plant Commun. 2021 Jun 12;2(5):100212. doi: 10.1016/j.xplc.2021.100212 (PMC8555439; doi:10.1016/j.xplc.2021.100212)
Supplement: Document S1. Supplemental Figures 1 and 2 and Supplemental Tables 1 and 2 [file mmc1.pdf]

Plant Communications, Volume *2*

Supplemental information

Split-HaloTag imaging assay for sophisticated microscopy of protein–protein interactions *in planta*

Rieke Minner-Meinen, Jan-Niklas Weber, Andreas Albrecht, Rainer Matis, Maria Behnecke, Cindy Tietge, Stefan Frank, Jutta Schulze, Henrik Buschmann, Peter Jomo Walla, Ralf-R. Mendel, Robert Hänsch, and David Kaufholdt

Supplemental Information

Split-HaloTag® Imaging Assay for Sophisticated Microscopy of Protein-Protein Interactions *in planta*

Plant Communications

Rieke Minner-Meinen[1], Jan-Niklas Weber[1], Andreas Albrecht[2], Rainer Matis[2], Maria Behnecke[1], Cindy Tietge[1], Stefan Frank[1], Jutta Schulze[1], Henrik Buschmann[3], Peter Jomo Walla[2], Ralf-R. Mendel[1], Robert Hänsch[1,4]* and David Kaufholdt[1]

[1]Institut für Pflanzenbiologie, Technische Universität Braunschweig, Humboldtstrasse 1, D-38106 Braunschweig, Germany

[2]Institut für Physikalische und Theoretische Chemie, Technische Universität Braunschweig, Hagenring 30.023c, D-38106 Braunschweig, Germany

[3]Botany Department, Universität Osnabrück, Barbara Str. 11, 49076 Osnabrück Germany

[4]Center of Molecular Ecophysiology (CMEP), College of Resources and Environment, Southwest University, Tiansheng Road No. 2, 400715 Chongqing, Beibei District, P.R. China

*author for correspondence: Tel: +49-(0)531-391-5867
Fax: +49-(0)531-391-8128
E-Mail: r.haensch@tu-bs.de

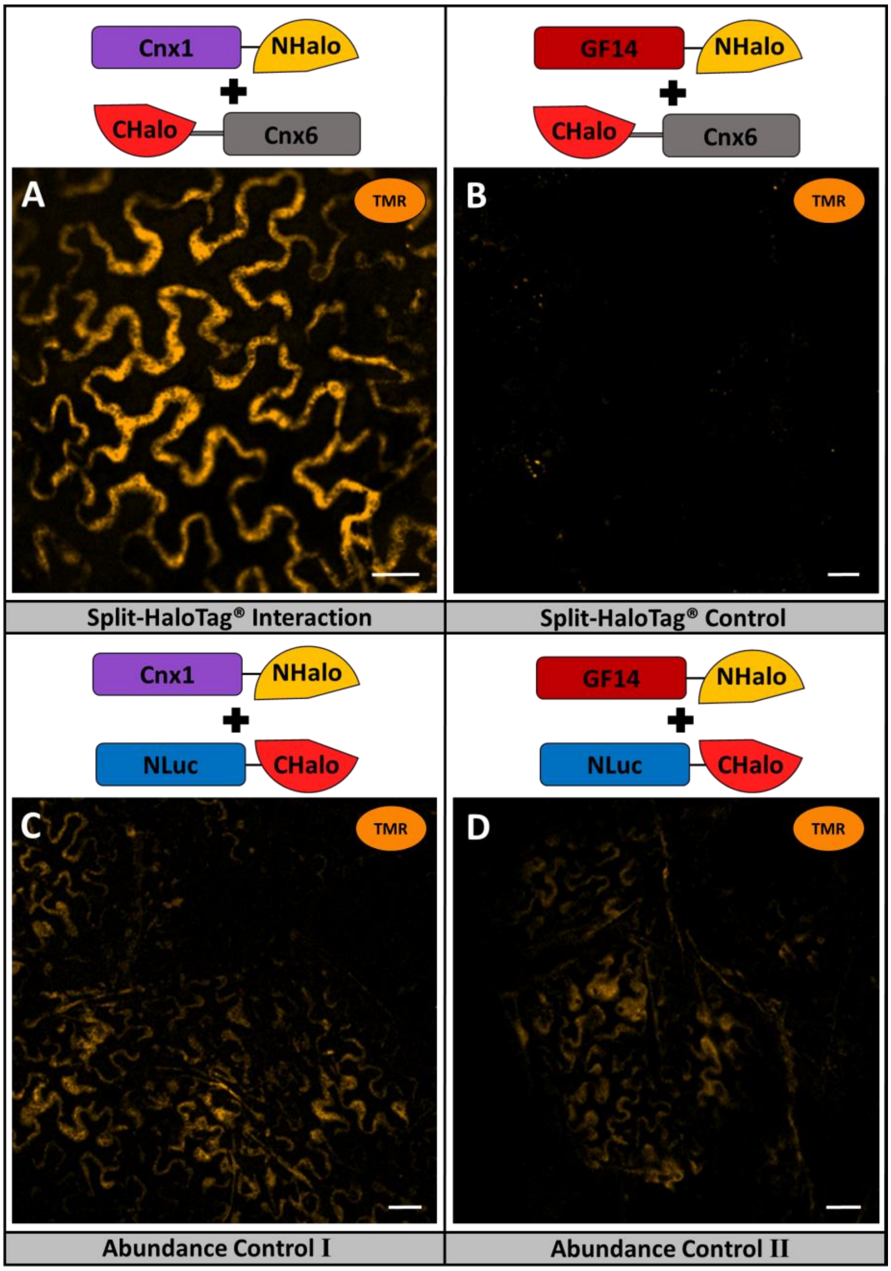

Fig. S1: Split-HaloTag® Protein-Protein Interaction Studies of Cnx1 and Cnx6 analogue to the BiFC study in Kaufholdt *et al*. (2013).

Shown are images of *N. benthamiana* epidermis cells via confocal microscopy of TMR. Staining of leaf discs was performed 5 days after transformation. All images were taken with identical setting (Plan-Neofluar 10x/0.3) for optimal comparison of fluorescence strength. Scale bars depict a length of 50 µm. **(A)** Interaction approach with Cnx1-NHalo and CHalo-Cnx6. **(B)** Negative control with the non-interacting proteins GF14-NHalo and CHalo-Cnx6. **(C/D)** Abundance control to validate negative control with **(C)** Cnx1-NHalo and NLuc-CHalo as well as **(D)** GF14-NHalo and NLuc-CHalo.

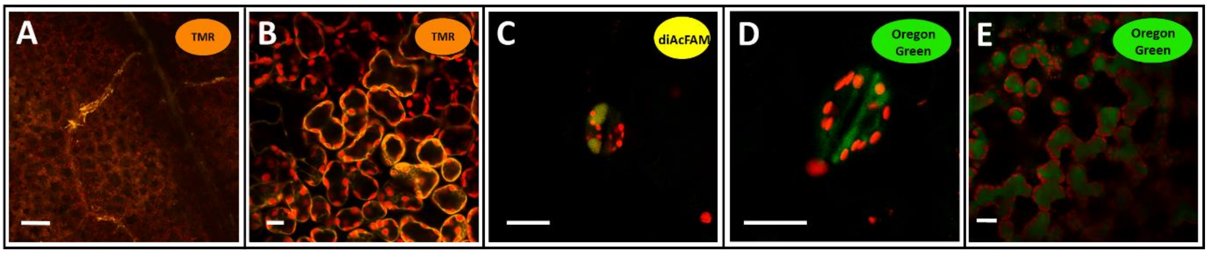

Fig. S2: Evaluation of HaloTag® fluorescent Ligands TMR, DiAcFAM and Oregon Green. Confocal microscopy of *N. benthamiana* leaf discs stained with TMR **(A/B)**, DiAcFAM **(C)** or Oregon Green **(D/E)**. Images were taken either with a Plan-Neofluar 10x/0.3 **(A)** or with a C-Apochromat 40x/1.2 water immersion objective **(B-E)**. Scale bars depict a length of 100 µm **(A)** or 20 µm **(B-E)**. **(A)** Unspecific TMR binding in vascular tissue. **(B)** Oversaturation of unbound TMR in parenchyma cells after using 2 µM dye. **(C/D)** Accumulation of unbound ligands in stomata after DiAcFAM **(C)** and Oregon Green **(D)** staining. **(E)** Accumulation of unbound Oregon Green in parenchyma cells.

Table S1: Primers for Cloning and sequencing of Split-HaloTag® related constructs and vectors. (**a**) Cloning of Split-HaloTag® Destination vectors (restriction + ligation); (**b**) Cloning of Split-HaloTag® Fusion Constructs. All oligonucleotides are purchased from Sigma-Aldrich (Steinheim, Germany).

Name	Sequence (5´-3´)	Purpose
**XbaI_ATG_Nhalo_for**	GGGGGGTCTAGAATGGGATCCGAAATCGGTACTG	a
**NHalo_ Linker_SpeI_rev**	CAAAAACTAGTGCCGCTGCCGCCGGTGCGGAAGGCCTGGAA GGTC	a
**XbaI_ATG_Chalo_for**	GGGGGGTCTAGAATGACCGACGTCGGCCGCAAGCTGAT	a
**CHalo_Linker_ SpeI_rev**	CCAAAAACTAGTGCCGCTGCCGCCACCGGAAATCTCCAGAGT AGAC	a
**XhoI_Linker_NHalo_f or**	GGGGGGCTCGAGGGCAGCGGCGGCATGGGATCCGAAATCG GTACTG	a
**NHalo_N_SacI_rev**	CCAAAAGAGCTCTTAGGTGCGGAAGGCCTGGAAGGTC	a
**XhoI_Linker_Chalo_ for**	GGGGGGCTCGAGGGCAGCGGCGGCACCGACGTCGGCCGCA AGCTG	a
**CHalo-N_SacI_rev**	CCAAAAGAGCTCTTAACCGGAAATCTCCAGAGTAGACAGCC	a
**AttB1_N-Halo_for**	GGGGACAAGTTTGTACAAAAAAGCAGGTTTAACCATTGGATCC GAAATCGGTACTGG	b (*Nhalo-cnx7)*
**Cnx7Ueb_Linker_ N-Halo-rev**	CAATCTTTGTAACTTCTTTGTCCATGCCGCCGCTGCCGGTGC GGAAGGCCTGGAAGGT	b (*Nhalo-cnx7)*
**N- Halo_Linker_Cnx7Ue b_ for**	ACCTTCCAGGCCTTCCGCACCGGCAGCGGCGGCATGGACAA AGAAGTTACAAAGATTG	b (*Nhalo-cnx7)*
**Cnx7_Stopp_AttB2_ rev**	GGGGACCACTTTGTACAAGAAAGCTGGGTCTCAGCCGCCGCT TATCGGAGGTAT	b (*Nhalo-cnx7)*
**AttB1_C-Halo_for**	GGGGACAAGTTTGTACAAAAAAGCAGGCTTAACCATGACCGA CGTCGGCCGCAAGCTGA	b (*Chalo-cnx6*, *Chalo-map65)*
**Cnx6Ueb_Linker_ C-Halo_rev**	GGTTCTTCTCCTCTGCAGACATGCCGCCGCTGCCACCGGAAA TGTCCAGAGTAGACAGC	b *(CHalo-cnx6)*
**C- Halo_Linker_Cnx6Ue b_ for**	GCTGTCTACTCTGGAGATTTCCGGTGGCAGCGGCGGCATGTC TGCAGAGGAGGAGGACC	b (*CHalo-cnx6)*
**Cnx6_Stopp_attB2_r ev**	GGGGACCACTTTGTACAAGAAAGCTGGGTCTCAAGAAGAAGA TTTGTTATCTCCTGTAAT	b (*CHalo-cnx6)*
**Map65- 3+4_Stopp_attB2_re v**	GGGGACCACTTTGTACAAGAAAGCTGGGTCTCATGGTGAAGC TGGAACTTGATG	b (*CHalo-map65*)
**Map65-3+4- Ueb_Link_C- Halo_rev**	CATGATTCTCTCACGAGCAGAGGCAGCGGCGGCACCGGAAA TCTCCAGAGTAGACAGC	b (*CHalo-map65*)
**C-Halo- Ueb_Link_Map65- 3+4_for**	GCTGTCTACTCTGGAGATTTCCGGTGCCGCCGCTGCCTCTGC TCGTGAGAGAATCATG	b (*CHalo-map65*)

Table S2: **Split-HaloTag® Destination and Expression vectors used within this work.**

Vector	Reporter	Fusion orientation	Selectable with
**pDest-*Nhalo-GW***	HaloTag® (N-Terminus)	N-terminal	Kanamycin/Chloramphenicol
**pDest-*Chalo-GW***	HaloTag® (C-Terminus)	N-terminal	Kanamycin/Chloramphenicol
**pDest-*GW-Nhalo***	HaloTag® (N-Terminus)	C-terminal	Kanamycin/Chloramphenicol
**pDest-*GW-Chalo***	HaloTag® (C-Terminus)	C-terminal	Kanamycin/Chloramphenicol
**pExp-*Nhalo-cnx7***	HaloTag® (N-Terminus)	N-terminal	Spectinomycin
**pExp-*Chalo- cnx6***	HaloTag® (C-Terminus)	N-terminal	Spectinomycin
**pExp-*Chalo-map65***	HaloTag® (C-Terminus)	N-terminal	Spectinomycin
**pExp-*cnx1-Nhalo***	HaloTag® (N-Terminus)	C-terminal	Kanamycin
**pExp-*la-Nhalo***	HaloTag® (N-Terminus)	C-terminal	Kanamycin
**pExp-*ckl6-Nhalo***	HaloTag® (N-Terminus)	C-terminal	Kanamycin
**pExp-*la-Chalo***	HaloTag® (C-Terminus)	C-terminal	Kanamycin
**pExp-*abd2-Chalo***	HaloTag® (C-Terminus)	C-terminal	Kanamycin
**pExp-*Nluc-Chalo***	HaloTag® (C-Terminus)	C-terminal	Kanamycin
**pExp-*cnx1-vyne***	Venus (N-Terminus)	C-terminal	Kanamycin
**pExp-*abd2-scyce***	SCFP (C-Terminus)	C-terminal	Kanamycin
**pExp-*la-scyce***	SCFP (C-Terminus)	C-terminal	Kanamycin
