# Supplementary material for: Split-HaloTag imaging assay for sophisticated microscopy of protein–protein interactions in planta
Source: Plant Commun. 2021 Jun 12;2(5):100212. doi: 10.1016/j.xplc.2021.100212 (PMC8555439; doi:10.1016/j.xplc.2021.100212)
Supplement: Document S2. Article plus supplemental information [file mmc2.pdf]

Plant Communications
Resource Article

CellPress
Partner Journal

Split-HaloTag imaging assay for sophisticated microscopy of protein–protein interactions *in planta*

Rieke Minner-Meinen[1], Jan-Niklas Weber[1], Andreas Albrecht[2], Rainer Matis[2], Maria Behnecke[1], Cindy Tietge[1], Stefan Frank[1], Jutta Schulze[1], Henrik Buschmann[3], Peter Jomo Walla[2], Ralf-R. Mendel[1], Robert Hänsch[1,4,*] and David Kaufholdt[1]

[1]Institut für Pflanzenbiologie, Technische Universität Braunschweig, Humboldtstrasse 1, 38106 Braunschweig, Germany

[2]Institut für Physikalische und Theoretische Chemie, Technische Universität Braunschweig, Hagenring 30.023c, 38106 Braunschweig, Germany

[3]Botany Department, Universität Osnabrück, Barbara Strasse 11, 49076 Osnabrück, Germany

[4]Center of Molecular Ecophysiology (CMEP), College of Resources and Environment, Southwest University, Tiansheng Road No. 2, Beibei District, 400715 Chongqing, P.R. China

***Correspondence: Robert Hänsch (r.haensch@tu-bs.de)**

https://doi.org/10.1016/j.xplc.2021.100212

ABSTRACT

An ever-increasing number of intracellular multi-protein networks have been identified in plant cells. Split-GFP-based protein–protein interaction assays combine the advantages of *in vivo* interaction studies in a native environment with additional visualization of protein complex localization. Because of their simple protocols, they have become some of the most frequently used methods. However, standard fluorescent proteins present several drawbacks for sophisticated microscopy. With the HaloTag system, these drawbacks can be overcome, as this reporter forms covalent irreversible bonds with synthetic photostable fluorescent ligands. Dyes can be used in adjustable concentrations and are suitable for advanced microscopy methods. Therefore, we have established the Split-HaloTag imaging assay in plants, which is based on the reconstitution of a functional HaloTag protein upon protein–protein interaction and the subsequent covalent binding of an added fluorescent ligand. Its suitability and robustness were demonstrated using a well-characterized interaction as an example of protein–protein interaction at cellular structures: the anchoring of the molybdenum cofactor biosynthesis complex to filamentous actin. In addition, a specific interaction was visualized in a more distinctive manner with subdiffractional polarization microscopy, Airyscan, and structured illumination microscopy to provide examples of sophisticated imaging. Split-GFP and Split-HaloTag can complement one another, as Split-HaloTag represents an alternative option and an addition to the large toolbox of *in vivo* methods. Therefore, this promising new Split-HaloTag imaging assay provides a unique and sensitive approach for more detailed characterization of protein–protein interactions using specific microscopy techniques, such as 3D imaging, single-molecule tracking, and super-resolution microscopy.

Key words: advanced microscopy, cytoskeleton, photostable fluorescent dyes, gateway cloning, molybdenum cofactor biosynthesis complex, protein–protein interaction, Split-HaloTag imaging assay

Minner-Meinen R., Weber J.-N., Albrecht A., Matis R., Behnecke M., Tietge C., Frank S., Schulze J., Buschmann H., Walla P.J., Mendel R.-R., Hänsch R., and Kaufholdt D. (2021). Split-HaloTag imaging assay for sophisticated microscopy of protein–protein interactions *in planta*. Plant Comm. **2**, 100212.

INTRODUCTION

An ever-increasing number of protein networks have been identified in plants (Zitnik et al., 2019). Therefore, understanding the cellular biology of substrate channeling pathways requires the characterization of protein–protein interactions (PPIs) in their native environment. A broad spectrum of *in vivo* methods have been employed to analyze PPIs; these include bimolecular fluorescence complementation (BiFC), which belongs to the

Published by the Plant Communications Shanghai Editorial Office in association with Cell Press, an imprint of Elsevier Inc., on behalf of CSPB and CEMPS, CAS.

This is an open access article under the CC BY license (http://creativecommons.org/licenses/by/4.0/).

group of protein fragment complementation assays (Struk et al., 2019). Basically, two non-fluorescent reporter fragments of a fluorescent protein (FP) are fused genetically to putative interaction partners, and an interaction between the two allowed formation of a bimolecular fluorescent complex (Kerppola, 2013). Consequently, BiFC enables not only detection of PPIs but also visualization and localization of the protein complex (Bhat et al., 2006). Furthermore, the use of FPs results in fluorescence signals without the invasive insertion of exogenous chemical compounds into the cell. Because of its simple protocols, BiFC has become one of the most popular and frequently used methods to study PPIs in plant cells (Kudla and Bock, 2016).

Conventional light and fluorescence microscopy is diffraction limited to a resolution of approximately 200 nm in the lateral (*x*–*y*) direction and about 600 nm in the axial (*z*) direction (Cremer and Masters, 2013). Many subcellular structures are smaller, which hampers their detailed observation (Huang et al., 2009). To circumvent these restrictions, advanced fluorescence imaging methods such as single-molecule detection, subdiffractional polarization imaging, or super-resolution microscopy have been developed to improve resolution and enable the study of molecular processes in more detail (Moerner and Kador 1989; Orrit and Bernard, 1990; Bode et al., 2008; Holleboom and Walla, 2014; Liao et al., 2010, 2011; Godin et al., 2014; Loison et al., 2018; Camacho et al., 2019). However, for such advanced imaging techniques, fluorescent dyes with high stability and brightness are needed (Banaz et al., 2018); these properties are hard to obtain from standard FPs, which show low quantum efficiency, blinking behavior, a high photobleaching rate during long-term observations (Reck-Peterson et al., 2006), and photoswitching (Morisaki and McNally, 2014), as well as the tendency to form oligomers (Miyawaki et al., 2003).

Self-labeling enzyme tags such as the HaloTag have been shown to overcome these drawbacks of FPs and are suitable for such microscopy methods and super-resolution imaging (Grimm et al., 2015; Lee et al., 2010). The HaloTag system (Promega, https://www.promega.de/) is based on the bacterial haloalkane dehalogenase DhaA (EC 3.8.1.5) from *Rhodococcus rhodochrous* (Liss et al., 2015). The tag was modified to form covalent irreversible bonds with synthetic chloroalkane ligands (Los et al., 2008; England et al., 2015). This covalent bond is formed rapidly in physiological environments and remains intact even under stringent conditions (Los and Wood, 2007). In addition, HaloTag proteins keep their monomeric structure, so the tag will not lead to oligomerization of protein fusion partners (Banaz et al., 2018). Numerous ligands are available for the HaloTag, including different fluorescent dyes with extended spectral range, photostability, and membrane permeability; these include the red fluorescent rhodamine derivative TMR (tetramethylrhodamine), the green fluorescent Oregon Green, and the yellow fluorescent diacetyl derivative of fluorescein DiAcFAM. Furthermore, dyes can be varied in their dosages to label either all or only a few molecules, which is necessary for single-molecule tracking approaches. Reck-Peterson et al. (2006), for example, used HaloTag and a TMR ligand to successfully label dynein in sea urchin axonemes and tracked single molecules with a precision of a few nanometers to reveal dynein's stepping behavior at microtubules. Moreover, several organic fluorophores were recently developed with photoactivatable properties especially for live-cell labeling and subsequent imaging (Lee et al., 2010), as well as with improved quantum efficiency and superior brightness while retaining excellent cell permeability (Grimm et al., 2015).

In 2012, Ishikawa and colleagues identified several split points within the HaloTag protein and demonstrated its reconstitution ability. These results gave rise to the idea of establishing the Split-HaloTag imaging assay *in planta*, as the usability of the HaloTag imaging system in plants had been shown previously (Lang et al., 2006). This new Split-HaloTag approach is particularly useful for characterizing the assembly of protein complexes at structural elements, including cell membranes or the cytoskeleton. Use of the above-mentioned microscopy techniques will enable us to study the local formation of a given complex with improved details compared with BiFC and conventional confocal laser scanning microscopy. Single-molecule tracking approaches with low concentrations of fluorescent dye will enable the tracing of complex mobility at the cytoskeleton or inside the membrane system. In this study, we use the previously described anchoring of the molybdenum cofactor biosynthesis complex via molybdenum insertase Cnx1 to filamentous actin (Kaufholdt et al., 2017) to establish the new Split-HaloTag imaging assay. In this way, we demonstrate the advantages of this assay for the imaging of PPIs *in vivo* via advanced microscopy.

RESULTS AND DISCUSSION

The Split-HaloTag constructs created in this study are based on the enhanced HaloTag-7 sequence, which has been optimized and improved with regard to solubility, stability, binding kinetics, and access to an optional TEV-cleavage site (Ohana et al., 2009). According to the initial experiment of Ishikawa and colleagues (2012), HaloTag protein was split at position 155/156 aa into the N-terminal fragment "NHalo" (aa 1–155) and the C-terminal fragment "CHalo" (aa 156–298). A stable bond between the HaloTag protein and its ligand is formed by the catalytic amino acid Asp^{106} in the NHalo fragment. In the wild-type dehalogenase, the nearby His^{272} would catalyze the hydrolysis of the intermediate, resulting in product release and enzyme regeneration (Los et al., 2008). In the mutated HaloTag protein, however, the substituted Asn^{272} (HaloTag-7) in CHalo traps the reaction intermediate as a stable covalent adduct. Taking human embryonic kidney cells as a model system, Ishikawa et al. (2012) demonstrated the general feasibility of Split-HaloTag reconstitution using a self-associating split-GFP system. They also monitored membrane fusion, as cell fusion enabled functional HaloTag reconstitution, resulting in TMR signals after staining. In this method paper, we set out to (i) examine the utility of the Split-HaloTag system as a new tool for studying PPIs in plant cells, (ii) develop staining and washing protocols that minimize background fluorescence, and (iii) demonstrate application examples of Split-HaloTag for sophisticated imaging and advanced microscopy methods.

Reconstitution of Split-HaloTag *in planta*

The cDNAs of the interaction partners were fused N- or C-terminally to the NHalo and CHalo fragments via fusion PCR and

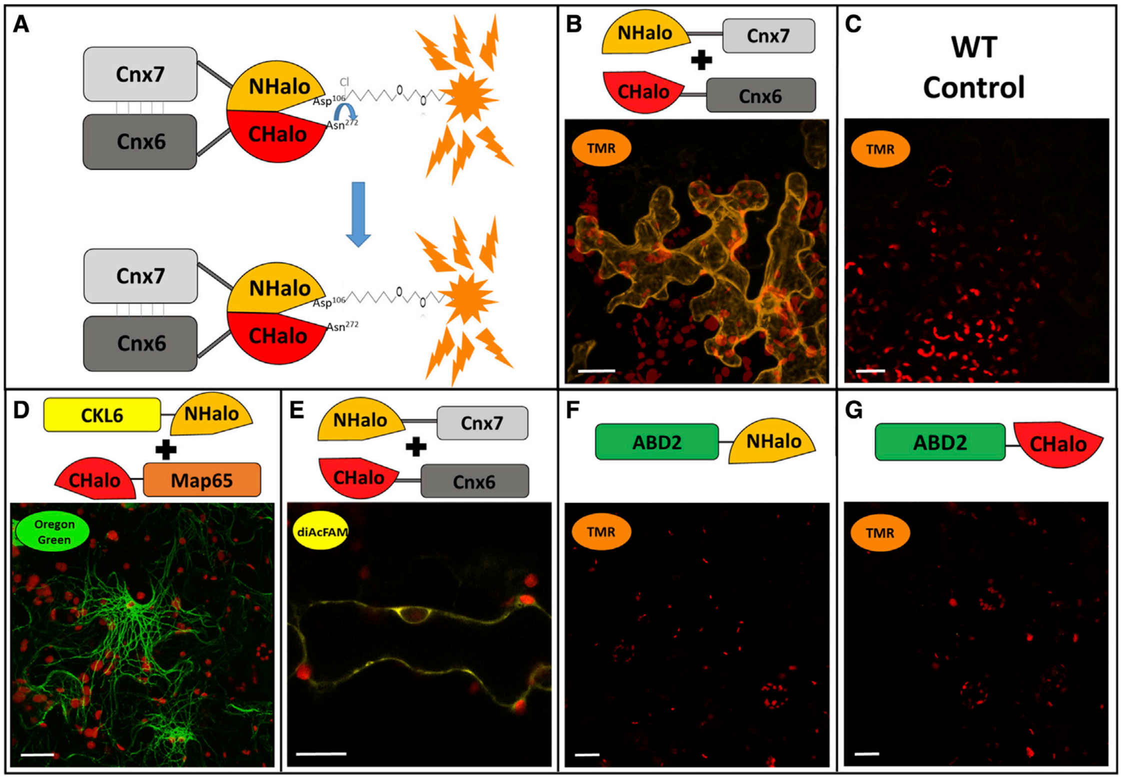

Figure 1. Testing Split-HaloTag complementation upon protein interactions of the MPT synthase complex with different fluorescent ligands

Shown are images of *N. benthamiana* epidermal cells obtained by confocal microscopy. Staining of leaf discs was performed 4–5 days after transformation. All images were taken with a C-Apochromat 40×/1.2 water-immersion objective. Scale bars represent 20 μm.

(A) Schematic illustration of Split-HaloTag reconstitution guided by the MPT synthase subunits Cnx6 and Cnx7. The important amino acids of each reporter terminus are depicted (Asp^{106} binds the linker, and Asn^{272} traps the catalytic reaction in a covalent binding stage).

(B) Cytosolic TMR fluorescence after transformation with NHalo-Cnx7 and Cnx6-CHalo.

(C) Negative control of a wild-type (WT) leaf after staining.

(D) Oregon Green fluorescence at microtubule filaments after transformation with microtubule binding constructs CKL6-NHalo and CHalo-Map65.

(E) Cytosolic DiAcFAM fluorescence after transformation with NHalo-Cnx7 and Cnx6-CHalo.

(F and G) Negative controls of single transformed Split-HaloTag reporter constructs fused to ABD2. **(F)** NHalo and ABD2, **(G)** CHalo and ABD2.

gateway cloning (see Material and methods). For high flexibility, Split-HaloTag GATEWAY-compatible destination vectors were generated, which enabled a fast and easy cloning of expression vectors with coding sequences of different proteins of interest (POIs). The formation of the heterotetrameric molybdopterin synthase (MPT) complex consiting of the subunits Cnx6 and Cnx7 of *Arabidopsis thaliana* was used to demonstrate reconstitution of the Split-HaloTag *in planta*. These proteins were chosen as the positive control pair because of their verified high binding strength (Kaufholdt et al., 2013). Interaction between the two proteins (Figure 1A) will bring the two HaloTag reporter fragments into close spatial proximity, thereby guiding the reconstitution of functional HaloTag proteins.

After the transformation of *Nicotiana benthamiana* epidermal cells and the staining with the fluorescent ligand TMR, specific cytosolic fluorescence was observed as a thin layer at the cell periphery (Figure 1B). Wild-type leaves were stained similarly, and the lack of fluorescence signals indicated that the washing steps were sufficient to remove unbound ligands (Figure 1C). Because the MPT synthase complex is localized in the cytoplasm, the HaloTag fragments CHalo and NHalo were capable of reconstitution, guided by the strong interaction of Cnx6 and Cnx7. The reconstituted HaloTag was able to bind the red fluorescent ligand TMR (Em_{max} 578 nm), the green fluorescent ligand Oregon Green (Em_{max} 520 nm) (Figure 1D), and the yellow fluorescent ligand DiAcFAM (Em_{max} 526 nm) (Figure 1E), even though DiAcFAM signals were weaker than TMR and Oregon Green signals when stained at similar concentrations. This demonstrates that the reconstituted HaloTag regained its function as a modified haloalkane dehalogenase to covalently bind HaloTag ligands. The three tested fluorescent ligands TMR, Oregon Green, and DiAcFAM can be seen as examples of the wide variety of available HaloTag ligands. As the two amino acids important for covalent ligand binding (Asp^{106} in NHalo and Asn^{272} in CHalo) are located on each of the two separate Split-HaloTag fragments, individual expression of NHalo or CHalo fragments alone will not enable ligand binding, which is a fundamental aspect of using Split-HaloTag imaging for the investigation of PPIs (Figure 1F and 1G).

After demonstrating general HaloTag reconstitution with a protein pair that forms a permanent complex, this new assay was tested in

a second approach using the MPT synthase subunit Cnx6 and molybdenum insertase Cnx1. In contrast to the permanent interactions within the MPT synthase complex (Cnx6/Cnx7), the interaction strength of the protein pair Cnx1/Cnx6 was previously found to be distinct but of a more transient nature (Kaufholdt et al., 2013). As in previous BiFC and split-luciferase experiments (Kaufholdt et al., 2013), we again conducted a full PPI study, including all necessary controls (see Material and methods). The cytosolic proteins NLuc (N terminus of the luciferase from *Photinus pyralis*) and G-box protein GF14 (AT1G78300) were used as control proteins. Both proteins show no interaction with Cnx1 or Cnx6. To ensure staining with equal TMR concentrations and a similar washing procedure, leaf discs for the interaction approach and controls were punched in slightly different sizes and stained simultaneously in the same syringe. The interaction approach showed strong cytosolic fluorescence (Supplemental Figure 1A), whereas the negative control and both abundance controls showed only weak cytosolic TMR signals (Supplemental Figure 1B–1D). A negative control without any fluorescence would be an unrealistic event; observed spontaneous self-assembly was expected, as it is typical for split-protein assays such as BiFC and split-luciferase if proteins are overexpressed in the small cytosolic space of plant cells (Gehl et al., 2009, 2011). Abundance controls are used to evaluate whether differences between the interaction approach and the negative control are due to differences in protein concentration. It can be concluded that the stronger signals observed in the interaction approach were not caused by a lower abundance of control proteins but were the result of Cnx1/Cnx6 interaction. Taken together, these results demonstrate that Split-HaloTag reconstitution also enables the investigation of transient interactions, and random self-assembly can be distinguished successfully from real interactions. Using the well-known transient interaction of the protein pair Cnx1/Cnx6 as an example, we validated the Split-HaloTag assay as an extension of the toolbox for PPI investigation *in planta*.

Insights into the staining protocol

Lang et al. (2006), who first introduced the HaloTag system to plant cells, attributed great importance to washing procedures to reduce non-specific background fluorescence, as background-free staining is often more complicated in plants than in animals. For interaction studies, comparison of fluorescence intensity and fluorescence patterns is the main task, and each form of background will falsify the result. However, after using the published destaining protocol, an excess of unbound dye remained in the tissue. Therefore, optimized staining and destaining procedures had to be established for using Split-HaloTag as a reliable tool for PPI studies. To improve staining protocols and identify the best HaloTag ligand for *in planta* PPI studies, several different factors were investigated: (i) the size of the analyzed leaf discs (6, 8, 10, and 12 cm in diameter), (ii) the concentration of ligands (0.25, 0.5, 1, and 2 μM), (iii) the ligand incubation time (1 min to 1 h), (iv) the number of subsequent washing steps (6 to 12), (v) the incubation time in washing solution (3 h to 12 h), and (vi) the aeration of the leaf discs in washing solution.

During this optimization process, several observations were made that are worth mentioning and must be considered to prevent misinterpretation and to obtain reliable interaction results. After TMR staining, fluorescence was always detected in the vascular tissue of both transformed and wild-type leaves, suggesting a non-specific adhesion between TMR and molecules in leaf veins (Supplemental Figure 2A). Therefore, leaf area farther away from vascular tissues should be used for analysis. Furthermore, staining with more than 0.5 μM TMR combined with an insufficient number of washing steps resulted in oversaturation and accumulation of unbound dye in the cytoplasm of parenchyma cells (Supplemental Figure 2B). This amount of unbound TMR accumulation increased when using larger or damaged leaf discs or older plants. Moreover, the recycling of frozen TMR solution led to non-specific aggregations inside the cells.

DiAcFAM staining produced an overall weaker fluorescence signal than TMR, but no staining of vascular tissue was observed (not shown). However, weak DiAcFAM signals (Em_{max} 521 nm) could easily be mistaken for typical plant background fluorescence at approximately 530 nm. Furthermore, accumulation of unbound ligands occurred, especially in stomata, after DiAcFAM (Supplemental Figure 2C) but also after Oregon Green (Supplemental Figure 2D) staining. In addition, Oregon Green accumulated inside the vacuoles of parenchyma cells if they were incubated for more than a few seconds in staining solution (Supplemental Figure 2E).

After testing the different staining parameters, optimal results for TMR staining were obtained using leaf discs 6–8 mm in diameter, a final TMR concentration of 0.5 μM in 2 ml fresh staining solution, 15 min incubation time, eight subsequent washing steps into a 20-ml syringe, and overnight incubation in washing solution. Samples were incubated with 10 ml washing solution by closing the screw lid and moving the plunger up and down approximately 10 times. Immediately before microscope analysis, two more washing steps were performed. Overnight incubation on a tumbling shaker or a rotary tube mixer was equally sufficient for all dyes, as long as there was sufficient air in the syringe to allow leaf disc aeration. It must be noted that the application of strong pressure during the staining and washing procedures can cause severe stress and damage to the cells. Therefore, pressure on leaf discs in the syringe should be as low as possible but just enough for the successful removal of all unbound ligands.

Oregon Green showed optimal results if a 0.5 μM staining solution was exchanged with 2 ml washing solution immediately after infiltration and incubated for 15 min. Then, the washing procedure was applied as described for TMR. This reduced but did not completely eliminate Oregon Green accumulation.

All three exemplarily tested dyes were suitable for intracellular labeling of HaloTag proteins. However, in this study, TMR showed the best applicability for studying cytosolic PPIs in a Split-HaloTag complementation assay including controls in *N. benthamiana* leaf discs. When analysis of confirmed interactions was desired, both TMR and Oregon Green, despite its accumulation, showed ideal properties for confocal laser scanning microscopy. Owing only to a different experimental setup, Oregon Green was used in super-resolution by polarization demodulation (SPoD) microscopy. It can be assumed that other HaloTag dyes will be suitable for other individual applications,

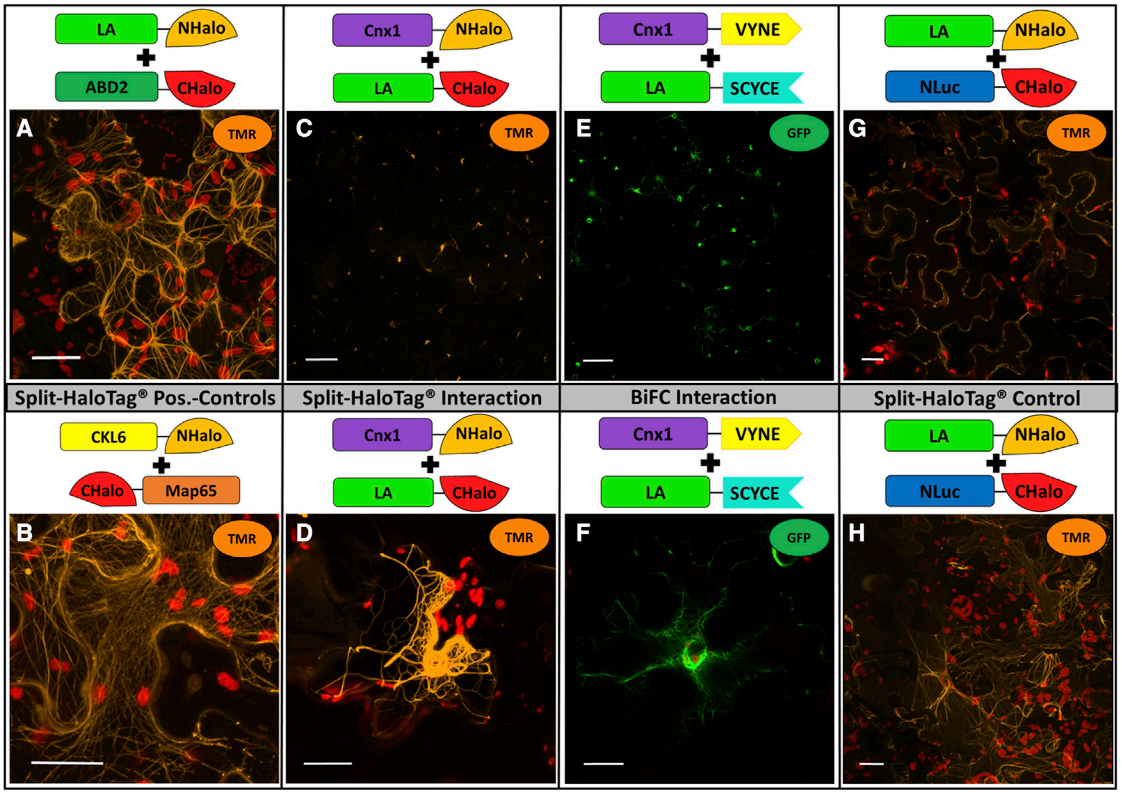

Figure 2. Split-HaloTag protein–protein interaction studies of Cnx1 and actin filaments via Lifeact
Shown are images of *N. benthamiana* epidermal cells obtained by confocal microscopy of TMR or GFP. Staining of leaf discs was performed 4–5 days after transformation. All images were taken with a Plan-Neofluar 10×/0.3 **(C, E, and G)** or C-Apochromat 40×/1.2 water-immersion objective **(A, B, D, F, and H).** Scale bars represent 100 **(C, E, and G)** or 20 μm **(A, B, D, F, and H)**.
(A) TMR fluorescence at actin after transformation with actin binding constructs LA-NHalo and ABD2-CHalo.
(B) TMR fluorescence at microtubules after transformation with microtubule binding constructs CKL6-NHalo and CHalo-Map65.
(C and D) Split-HaloTag approach with Cnx1-NHalo and LA-CHalo.
(E and F) BiFC approach with Cnx1-VYNE and LA-SCYCE.
(G and H) Corresponding Split-HaloTag negative control where Cnx1 was replaced by the independent protein NLuc. **(C–F)** were imaged with identical settings for optimal comparison of strength and pattern.

and disadvantages of one dye are advantageous in another experimental setting. For example, the photoinstability of DiAcFAM limits its use in quantitative fluorescent cell imaging but may be advantageous for fluorescence recovery after photobleaching and other applications in which photoinstability is required (Promega, 2018). As TMR also penetrates peroxisomal membranes (Lang et al., 2006) and the nuclear envelope (unpublished data), interaction studies in other organelles would also be possible.

Validation of Split-HaloTag imaging upon protein–protein interaction at cytoskeletal elements

To further demonstrate the utility of Split-HaloTag for *in planta* PPI studies, a third approach was conducted with proteins attached to cytoskeletal structures such as filamentous (F) actin and microtubules. Using this approach, it was possible to test whether the Split-HaloTag system enables the investigation of the assembly of different proteins at cytoskeletal elements. Both structures were not labeled directly to NHalo or CHalo termini, but instead via binding proteins, as fusion of larger reporter fragments directly to globular actin or tubulin proteins might disturb their polymerization processes. For F-actin labeling, the binding domains of fimbrin from *A. thaliana* (ABD2; Sheahan et al., 2004) and Abp140 from *Saccharomyces cerevisiae* (Lifeact/LA; Riedl et al., 2008) were used. For microtubule labeling, microtubule binding domains of the two proteins Casein-Kinase-1-Like-6 (CKL6; Ben-Nissan et al., 2008) and Microtubule-Associated Protein 65 (Map65; Hamada, 2007) from *A. thaliana* were investigated. The cytoskeleton binding proteins show no direct protein interactions with each other. However, their affinity and subsequent binding and anchoring to the cytoskeletal structures result in such spatial proximity that they are able to act as a model for a direct interaction of a cytoskeleton-associated protein complex (Kaufholdt et al., 2016a). Expression of F-actin binding protein constructs followed by HaloTag reconstitution and staining resulted in TMR-specific fluorescence visible as transversely arranged filaments with branches distributed throughout the cytoplasm (Figure 2A). The use of CLK6-NHalo and CHalo-Map65 to label microtubules upon Split-HaloTag reconstitution (Figures 1D and 2B) revealed filamentous structures more equally distributed throughout the cell with fewer cross bridges compared with actin filaments. These structures are typical for F-actin and microtubules, respectively, and were observed

previously in BiFC experiments (Kaufholdt et al., 2016a). In all interaction approaches, both actin filaments and microtubules could successfully be visualized upon the interaction of cytoskeletal binding proteins.

Next, we aimed to validate this new assay using the binding of molybdenum insertase Cnx1 to F-actin as a well-characterized example of a PPI at a cell structure (Kaufholdt et al., 2016a). This established setting with cytoskeleton binding proteins for *in vivo* interaction studies was used to investigate whether the Split-HaloTag system demonstrated F-actin binding of Cnx1 in the same manner as has previously been shown in BiFC experiments (Kaufholdt et al., 2016a). In the interaction approach, reporter fusion constructs of Cnx1 and the actin binding protein LA were co-expressed in *N. benthamiana*. For comparison, the BiFC approach described by Kaufholdt et al. (2016a) was included, using the reporter halves VYNE (N terminus of Venus) and SCYCE (C terminus of SCFP) (Gehl et al., 2009). The BiFC and Split-HaloTag complementation assays produced almost identical results (Figure 2C–2F). Both TMR and GFP fluorescence were detected in a filamentous pattern that was concentrated at the actin nucleus basket and thinned out at F-actin toward the cellular cortex. A typical pattern when studying actin-interacting protein complexes, which is reminiscent of a "starry sky" (Kaufholdt et al., 2016a), was observed in both approaches, caused by F-actin anchoring of the interacting protein in close proximity to its synthesis by the two actin binding domains of LA and Cxn1. However, TMR fluorescence in the negative control was equally distributed throughout the cell, and no starry sky was observed (Figure 2G and 2H). Consequently, identical results were observed with BiFC and Split-HaloTag; both showed the characteristic starry sky-like pattern demonstrating protein interaction with F-actin as discussed by Kaufholdt et al. (2016a). This provides an example of the applicability of the Split-HaloTag system for a protein that binds to the cytoskeleton.

Application examples of Split-HaloTag for sophisticated imaging

As conventional light and fluorescence microscopy are diffraction limited, advanced fluorescence imaging methods such as single-molecule detection, subdiffractional polarization imaging, or super-resolution microscopy have been developed to improve resolution and allow for more detailed study of molecular processes (Moerner and Kador 1989; Orrit and Bernard, 1990; Bode et al., 2008; Holleboom and Walla, 2014; Liao et al., 2010, 2011; Godin et al., 2014; Loison et al., 2018; Camacho et al., 2019). Such imaging techniques require stable fluorescent dyes that emit light at various wavelengths, which is difficult to achieve using standard FPs (Banaz et al., 2018). When BiFC is used to localize and image a specific protein interaction, the detected fluorescence intensity is directly related to fusion construct expression levels. By contrast, the Split-HaloTag system has the advantage of adjusting the dosage of fluorescent dyes customized for the individual application. Using 0.25 μM compared with 0.5 μM, for example, enabled us to observe the attachment of molybdenum insertase Cnx1 to F-actin in much greater detail (Figure 3A and 3B). Using even lower concentrations would label even fewer HaloTag proteins and enable tracking of the dynamic movements of single protein complexes.

The stability of fluorescent dyes is of great importance, especially during long-term observations. Although FPs have improved significantly in recent years with regard to their photon budget (Kubitscheck et al., 2000), standard FPs used for most BiFC experiments show low quantum efficiency, blinking behavior, and a high photobleaching rate (Reck-Peterson et al., 2006). In a direct comparison, the GFP used in the BiFC approach bleached much faster after 100 iterations (100% laser power) than did TMR with the same settings (Figure 3C–3F).

To demonstrate the stability and resolution potential of TMR for confocal laser scanning microscopy, the interaction of Cnx1 and the actin binding protein ABD2 was again used as an example. For this purpose, each layer of the cell must be scanned in very thin optical slices (micrometer range or less), and such detailed imaging can take several minutes. The stable TMR fluorescence of the Split-HaloTag enabled more defined results in the cell images (Figure 4A) compared with BiFC approaches (Figure 4B), as demonstrated by the very thin filaments of the F-actin network, which were barely detectable by BiFC.

Super-resolution Airyscan and structured illumination microscopy (SIM) corroborated these findings (Figure 4E–4J). Airyscan and linear SIM are able to double the wide-field resolution, and iterative SIM methods like SIM^2 can even quadruple it. When using the full width at half-maximum (FWHM) as a resolution criterion, measured by profiling actin filaments, we were able to obtain smallest measured diameters of 140 nm, 75 nm, and 150 nm for SIM, SIM^2, and Airyscan, respectively, using the Split-HaloTag with TMR label (Figure 4E, 4G, and 4I). However, the lower excitation wavelength FWHM data from BiFC produced values slightly higher or comparable to those of Split-HaloTag staining: 145 nm, 90 nm, and 150 nm (Figure 4F, 4H, and 4J). This is probably because the organic dye TMR has a higher quantum yield and hence provides a better signal-to-noise ratio than the GFP tag used for BiFC.

SPoD microscopy was used as another example to demonstrate the performance of the Split-HaloTag system. This advanced fluorescence imaging technique is a subdiffractional polarization imaging method that enables measurement of the average orientation of fluorescent dyes attached to different structures and was first described by Hafi et al. (2014). Fluorescent molecules are illuminated using linearly polarized light. This causes the fluorophores to be excited at different times and results in a modulated fluorescence intensity from the fluorophores. Depending on the orientation of the illuminated fluorophores (or, more specifically, the orientation of their transition dipole moments), the observed fluorescence intensity will be phase shifted, and differently oriented fluorophores will emit periodic signals that peak at different points in time. Therefore, analyses with deconvolution algorithms enable the high-resolution imaging of cell structures (for details see Albrecht et al., 2020). During each measurement, 2000 frames were recorded, which in itself is not problematic when using stable dyes. Split-HaloTag constructs with the two microtubule binding domains of CLK6 and Map65 were used for this purpose. Overexpression of MAP65 isotypes is known to result in microtubule bundling (Mao et al., 2006). After transformation and expression, leaf discs were stained with Oregon Green, the wavelength of which was more appropriate for SPoD

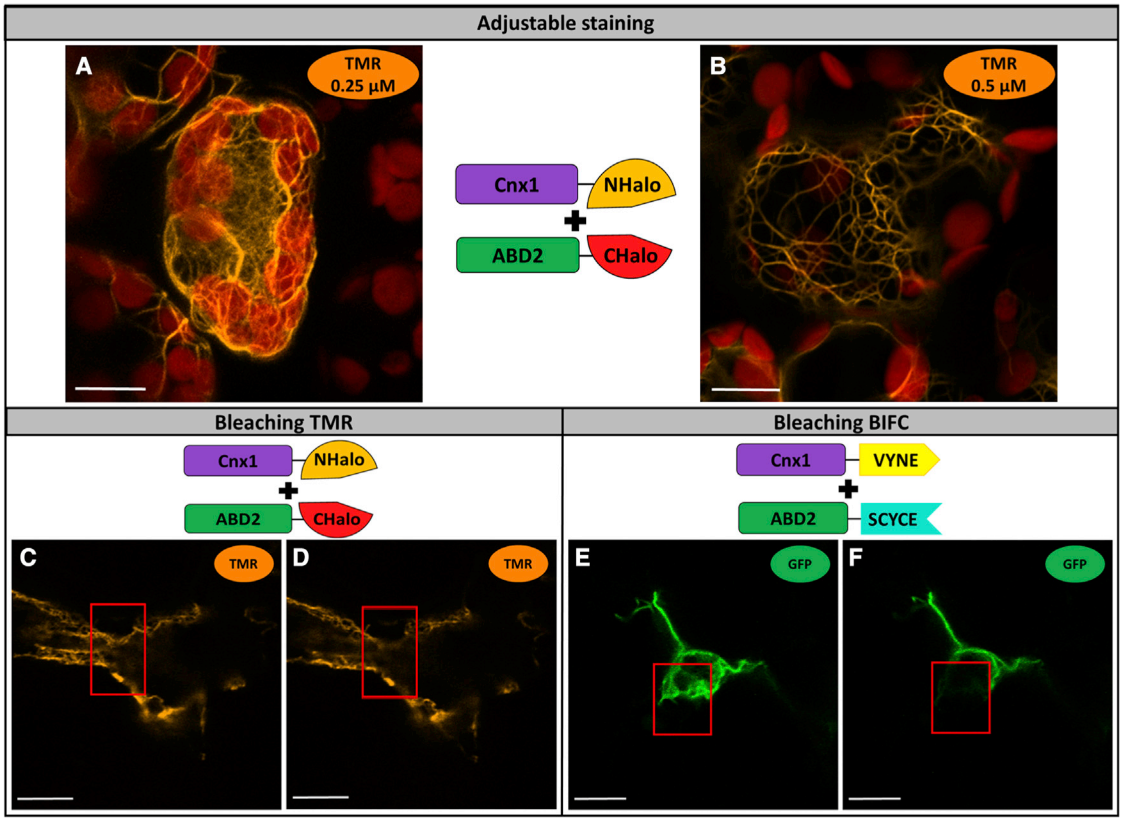

Figure 3. Advantages of Split-HaloTag imaging via confocal microscopy
Shown are representative *N. benthamiana* cells 4–6 days after transformation.
(A and B) Staining with different concentrations of TMR (**[A]** 0.25 µM and **[B]** 0.5 µM) to optimize fluorescence intensity.
(C–F) Bleaching experiments with **(C and D)** TMR and **(E and F)** BiFC. Shown are pictures before **(C and E)** and after **(D and F)** 100 iterations of 100% laser power in the marked section (red rectangle).

microscopy. The resulting fluorescence permitted the observation of individual microtubules (Figure 4C). Although high amounts of background fluorescence and out-of-focus signal complicated the recording and modulation analysis, sub-diffractional separation in a branching region of distinct fibers was observed in the deconvolved image (Figure 4D) and was supported by different phases as visualized by a simple red/green color code. These different phases in the branching region of the two fibers were already observable in the raw modulation data. Certainly, future work is needed to enhance separation by different phases in addition to pure image deconvolution for entire cell images. Nevertheless, the Split-HaloTag imaging assay can be used for such advanced fluorescence imaging techniques, which failed in the case of BiFC.

Comparison of Split-HaloTag imaging assay to other fluorescence-based methods

During the process of Split-HaloTag validation, a series of confirmed protein interactions was analyzed, demonstrating the utility of the method for *in planta* PPI investigation. Spontaneous self-assembly without protein interaction is one limitation of split-protein assays such as BiFC (Struk et al., 2019) and split-luciferase (Gehl et al., 2009/2011), and this also applies to Split-HaloTag. When proteins are overexpressed in the small cytosolic space of plant cells, reporter reconstitution obviously occurs merely by chance because of high protein abundance. Furthermore, BiFC fragments have an intrinsic affinity for each other, and once the BiFC complex is formed, this formation is irreversible (Kerppola, 2013). Intrinsic affinity of Split-HaloTag fragments has not been shown, but irreversibility can also be assumed for Split-HaloTag because ligands will covalently bind only to reconstituted HaloTag proteins. However, this non-reversible nature is also an advantage, as it enables the detection of weak or one-time interactions (Lampugnani et al., 2018). The spontaneous self-assembly of both BiFC and Split-HaloTag experiments can be overcome using abundance controls, which provide an adequate evaluation of whether differences between the interaction approach and the negative control are due to an interaction or merely to different protein concentrations (Kaufholdt et al., 2013). Therefore, the Split-HaloTag imaging assay is a similar valid method for *in planta* PPI studies.

With regard to interaction dynamics and temporal resolution, PPI methods such as split-luciferase (Gehl et al., 2011) and Foerster-resonance energy transfer (FRET) and its modifications, e.g., FRET–fluorescence-lifetime imaging microscopy (FRET-FLIM), have emerged as powerful approaches (Day and Davidson, 2012). In FRET, instead of a split reporter, the two interacting POIs are each fused to full-length FPs, and energy transfer

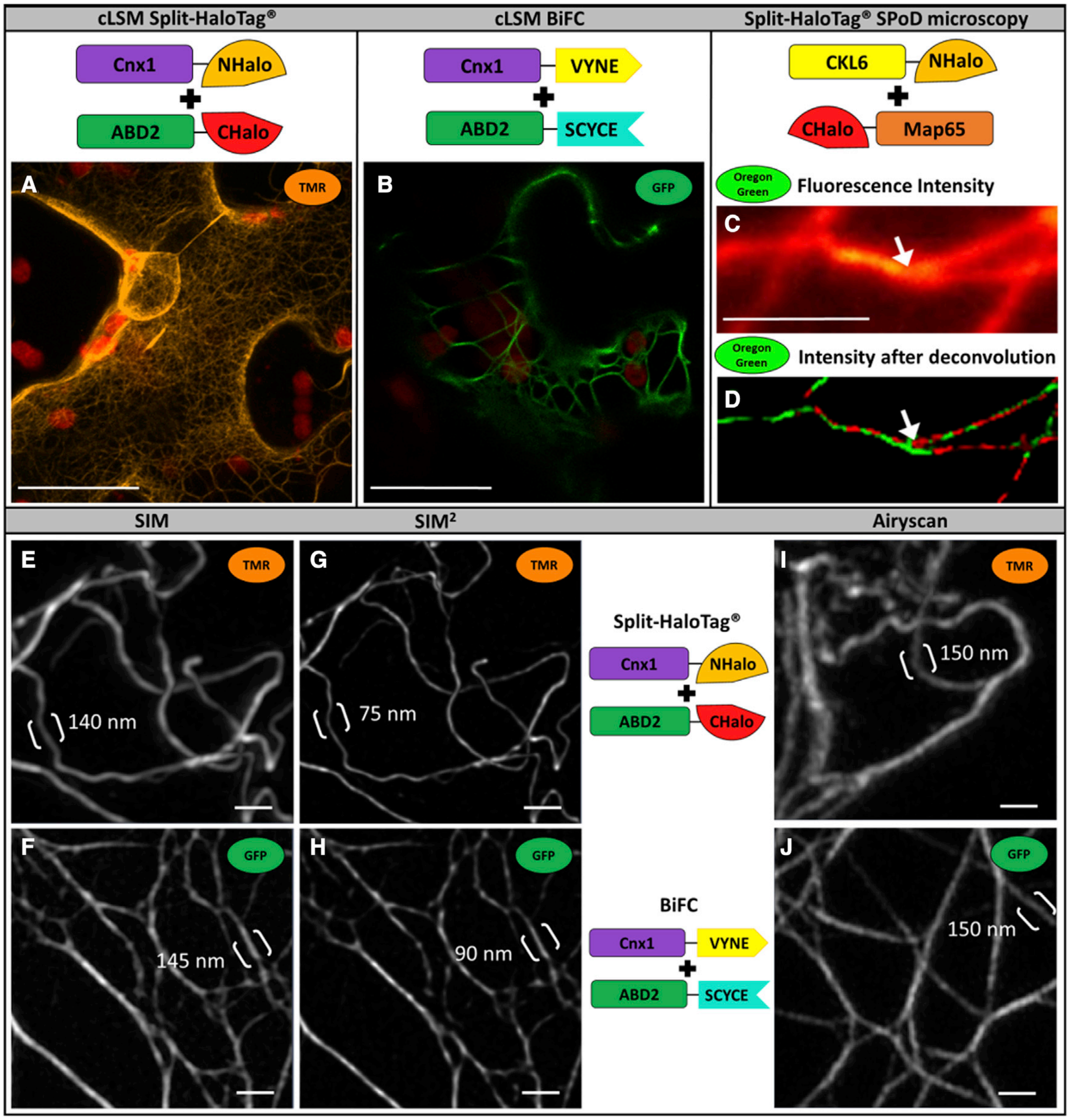

Figure 4. Analysis of Split-HaloTag images via confocal microscopy and super-resolution techniques
Shown are representative *N. benthamiana* cells 4–6 days after transformation.
(A and B) Interaction studies at actin filaments with Cnx1 and ABD2 via Split-HaloTag **(A)** or BiFC **(B)**. Split-HaloTag staining was performed with TMR. Images were taken with a C-Apochromat 40×/1.2 water-immersion objective. Scale bars represent 20 μm.
(C and D) SPoD microscopy of microtubules stained with Oregon Green after transformation of the Split-HaloTag microtubule binding constructs CKL6-NHalo and CHalo-Map65. **(C)** Diffraction-limited image depicting averaged raw fluorescence intensity. **(D)** Phase-color-coded fluorescence intensity image after 1000 iterations of the deconvolution algorithm. The red/green color code supports subdiffractional separation of the fibers at a distinct branching fork (arrow) that is not visible in the conventional diffraction of the wide-field image. However, future work is needed to enhance separation by different phases in addition to pure image deconvolution for entire cell images. The raw data scale bar represents 2 μm.
(E–H) Structured illumination and **(I and J)** Airyscan images of actin filaments labeled with Split-HaloTag **(E, G, and I)** and BiFC **(F, H, and J)**. Brackets indicate the area where profiles were taken, with the FWHM values displayed. Scale bar indicates 1 μm.

from donor to acceptor can occur only when both proteins are in close proximity (Struk et al., 2019). Measuring the fluorescence lifetime via FRET–FLIM enabled the study of cell-type-specific complex formation *in vivo* (Long et al., 2017). Like Split-HaloTag, FRET–FLIM shows its benefits with sophisticated microscopy systems (Wallrabe and Periasamy, 2005). However, it has higher requirements for advanced equipment, specialized software, and the expertise to process and evaluate the data (Lampugnani et al., 2018). Therefore, both methods can complement one another: FRET–FLIM can provide information on interaction dynamics, whereas Split-HaloTag can specify the localization, as it allows for single-molecule tracking approaches.

Regarding protocol simplicity and handling, the Split-HaloTag imaging assay cannot outcompete BiFC as the method of choice for studying putative protein interactions. Therefore, due to its simple

protocols, BiFC has become one of the most popular and frequently used methods for studying PPIs in plant cells (Kudla and Bock, 2016). The additional infiltration of a fluorescent ligand into the cell with subsequent washing steps was a disadvantage compared with BiFC. However, BiFC is less useful when specific confirmed protein interactions must be observed and imaged in greater detail. Furthermore, when the investigated interaction results in a characteristic structural pattern, it can be visualized very specifically and in great detail using the Split-HaloTag imaging assay with advanced fluorescence imaging techniques, which failed in the case of BiFC. All in all, the Split-HaloTag imaging assay fits perfectly into the pre-existing toolbox of methods; it bridges the gap between detection, localization, and visualization of PPIs, as it enables imaging of protein complexes at high resolution.

In this study, the Split-HaloTag imaging assay was established for the first time *in planta*. Vectors were cloned, and the reporter termini NHalo and CHalo were tested for reconstitution in both fusion orientations to the POI and in all four orientation combinations with each other. The applicability of the system for PPI studies was demonstrated using previously published protein interactions involved in the formation of the molybdenum cofactor biosynthesis complex and its anchoring to F-actin.

The benefit of the Split-HaloTag system lies in the ability to visualize specific confirmed protein interactions with advanced imaging techniques. Therefore, this system can be used in the future for sophisticated imaging techniques such as 3D microscopy, polarization microscopy, single-molecule tracking, or super-resolution imaging that require brighter and more stable fluorescent markers. Localization of protein complexes can be observed with the Split-HaloTag imaging assay in a distinct manner. In live-cell microscopy, the method combines *in vivo* split-reporter analyses with the previously shown advantages of the HaloTag: a large set of differently colored fluorescent ligands, their photostability compared with FPs, and the ability to vary labeling intensity by adjusting the dye dosage independent of protein expression. In recent years, improved FPs have already been used for split-reporter applications (Xie et al., 2017); however, some drawbacks still remain. Therefore, this Split-HaloTag imaging assay provides a unique and sensitive approach for the characterization of PPIs by combining all the advantages of the HaloTag system with the advantages of protein fragment complementation assays.

MATERIAL AND METHODS

Cloning of Split-HaloTag gateway destination vectors

The optimized HaloTag-7 sequence (298 amino acids) from Promega (https://www.promega.de/) was genetically split at position 155/156 aa into the N-terminal fragment NHalo (aa 1–155) and the C-terminal fragment CHalo (aa 156–298) according to the initial experiment of Ishikawa and colleagues (2012). The binary destination vectors pDest-*Cluc*-GW and pDest-GW-*Cluc* (Gehl et al., 2011) were used to create Split-HaloTag GATEWAY destination vectors and enable C-terminal Split-HaloTag reporter fusion. PCR primers were designed to fuse specific restriction enzyme recognition sequences at both reporter fragments (Supplemental Table 1). After amplification, *Cluc* fragments were exchanged by restriction digestion and ligation for *Nhalo* or *Chalo* residues using the restriction sites *Xba*I and *Spe*I (N-terminal) and *Xho*I/*Sac*I (C-terminal), respectively (restriction enzymes purchased from Thermo Fischer Scientific, https://www.thermofisher.com), to create pDest-*Nhalo*-GW, pDest-*GW-Nhalo*, pDest-*Chalo*-GW, and pDest-*GW-Chalo* (Supplemental Table 2).

Expression vectors

Coding sequences of Cnx6 (AT2G43760), Cnx7 (AT4G10100), and Map65 (amino acids 340–587; AT5G55230) were fused to Split-HaloTag fragments via a two-step fusion PCR with Phusion polymerase purchased from Thermo Fischer Scientific. For the first PCR, each single cDNA and reporter fragment was created with overlapping sequences, which enabled the assembly of fusion constructs (primers listed in Supplemental Table 1). For the second step, the products of the first PCR were assembled using the overlapping matching sequences and then amplified into a single fragment. This *att*B-site-flanked constructs were subcloned via BP reaction into the donor vector pDONR/Zeo to create entry vectors. Recombining these into pK7WG2 (Karimi et al., 2002) using LR reactions generated the expression vectors pExp-*Nhalo-cnx7*, pExp-*Chalo-cnx6*, and pExp-*Chalo-map65*.

All BiFC expression vectors and entry vectors with coding sequences of Cnx1 (AT5G20990), LA (Lifeact; amino acids 1–17 of the *S. cerevisiae* protein ABP140), ABD2 (amino acids 325–687; AT4G26700), CKL6 (amino acids 302–479; AT4G28540), and NLuc were available and are described by Kaufholdt et al. (2016a). The entry vectors were used to clone Split-HaloTag expression vectors via LR reactions into pDest-*GW-Nhalo* and pDest-*GW-Chalo* to create pExp-*cnx1-Nhalo*, pExp-*la-Nhalo*, pExp-*ckl6-Nhalo*, pExp-*la-Chalo*, pExp-*abd2-Chalo*, and pExp-*Nluc-Chalo*.

Plant transformation

N. benthamiana wild-type plants were cultivated in soil under greenhouse conditions. They were used for *Agrobacterium*-mediated transient transformation of fusion constructs 7–12 weeks after germination as described by Gehl et al. (2011). *Agrobacterium* strains C58C1/pMP90 carrying binary expression vectors were freshly grown (48 h at 28°C) on solid CPY medium (0.1% [w/v] yeast extract, 0.5% [w/v] casein peptone, 0.5% [w/v] sucrose, 2 mg/l $MgSO_4 \cdot 7H_2O$ [pH 7]; 1.5% [w/v] agar) containing rifampicin (50 mg/l), gentamycin (50 mg/l), and kanamycin (50 mg/l). Helper strain p19 (Voinnet et al., 2003) was grown on CPY medium containing rifampicin (50 mg/l) and kanamycin (50 mg/l). After growing for 20 h in 9 ml of liquid CPY at 200 rpm (28°C), cells were transferred into fresh activation medium (10 mM MES/KOH [pH 5.6], 10 mM $MgCl_2$, 150 μM acetosyringone). Before infiltration of the bacteria into the leaves, each strain was diluted in activation medium to an optical density of OD_{600} = 0.9 (final OD_{600} = 0.3). Then, three strains were mixed for each transformation: (i) one strain containing an NHalo construct, (ii) one strain containing a CHalo construct, and (iii) the helper strain p19. After incubation for 2 h at 50 rpm (28°C), mixed *Agrobacterium* suspensions were infiltrated into the abaxial side of young, fully expanded leaves. Plants were incubated for 3–5 days in the greenhouse.

Staining of *N. benthamiana* leaf discs with HaloTag ligands

The staining protocol was based on the work of Lang et al. (2006). Leaf discs (6–10 mm) of *N. benthamiana* leaves were transferred into a 20 ml syringe with a screw lid and infiltrated with 2–4 ml ligand solution (0.5, 1.0, or 2.0 μM TMR, DiAcFAM, or Oregon Green in 10 mM MES/KOH [pH 5.6] and 10 mM $MgCl_2$). All dyes were purchased from Promega. Syringes with leaf discs were wrapped in aluminum foil and incubated for 0.5, 15, 30, or 60 min on the work bench, on a tumbling shaker, or on a rotary tube mixer. After staining, the samples were washed with 10 ml washing solution by closing the screw lid and moving the plunger up and down 10 times. Washing steps were repeated with fresh washing solution 6–12 times. Furthermore, one duration before the last washing step of 0, 3, or 12 h in washing solution was performed.

Confocal laser scanning microscopy

The confocal laser scanning microscope LSM 510 Meta from Zeiss (Göttingen, Germany) was used. The cLSM 510 Meta scanhead was connected to the Axiovert 200M. All images were examined using either the Plan-Neofluar 10×/0.3 or the C-Apochromat 40×/1.2 water-immersion objective. For excitation, both an argon laser (488 nm for BiFC, Oregon Green, DiAcFAM, and chlorophyll fluorescence) and a helium–neon laser (543 nm line for TMR) were used. The emitted light passed the primary beam-splitting mirror UV/488/543/633 and was separated by a secondary beam splitter at 545 nm. Fluorescence was detected with filter sets as follows: BP 505–530 nm for BiFC (Em_{max} 515 nm), Oregon Green (Em_{max} 520 nm), and DiAcFAM (Em_{max} 521 nm); BP 560–615 for TMR (Em_{max} 578 nm); LP 650 nm for chlorophyll fluorescence. Bright-field images were taken with the transmitted light photomultiplier. All images were taken using ZEN 2009 Zeiss microscope software and processed with ZEN Lite and Fiji (Schindelin et al., 2012). The images shown depict representative cells of several analyzed leaves from at least three independent transformations.

Interaction studies

For both BiFC and Split-HaloTag interaction studies, several representative pictures were taken from 5–10 leaf discs of two or three plants per experiment with identical microscope settings for the interaction approach and the respective negative and abundance controls (Kaufholdt et. al., 2016b). For a full interaction study, the following combinations were tested:

I) A + B = interaction approach of the POIs
II) A + C1 = negative control
III) B + C2 = abundance control 1
IV) C1 + C2 = abundance control 2

C1 and C2 represent control proteins that show no interaction with POI A or B. Therefore, observed signals in the negative control originate from spontaneous reconstitution of the split-reporter halves. As another factor needed for a correct interpretation of PPI, abundance controls were employed to clarify whether POI B in the interaction approach is present in an amount corresponding to that of the negative control C1. Due to different regulation of protein expression or degradation, the number of proteins can vary, even if expression is controlled by the same promoter. To avoid misinterpretation of protein interactions due to different protein concentrations in the cell, an approach was performed with the fusion construct of interest B and a non-interaction construct C2, which carried the reporter fragment counterpart. In the other leaf half, the negative control construct C1 was coexpressed with C2. Both fusion constructs will reconstitute with this non-interacting construct just by chance and diffusion, dependent on their abundance in the cell. By doing so, the relative abundance levels of the POI and the negative control can be estimated under experimental conditions *in planta*.

Super-resolution microscopy by structured illumination microscopy

For SIM, the Zeiss ELYRA 7 with Lattice SIM system was employed. Images were acquired using the C-Apochromat 63×/1.2 NA Corr water objective. Excitation was performed with the 488 nm laser line at 2% AOTF setting for BiFC using a BP 495–550 nm emission filter and a camera exposure time of 20 ms. TMR was excited by the 561 nm laser line at 1.5% AOTF setting; an emission band-pass filter of 570–620 nm was used, and a camera exposure time of 15 ms was selected in this case. A *Z* stack of approximately 150 planes was obtained, and the stack was analyzed with the ZEN SIM processing tool using the SIM (linear Wiener filtering) or the SIM^2 (dual iterative deconvolution) module. SIM processing can double and SIM^2 processing can quadruple wide-field resolution. For FHWM measurements, profiles of tubular structures were analyzed.

Super-resolution by Airyscan microscopy

For Airyscan imaging, the Zeiss LSM 980 Airyscan 2 system was used in SR mode with the LD LCI Plan-Apochromat 40×/1.2 Imm Autocorr objective. Excitation was performed with the 561 nm and 488 nm laser lines at 0.2% AOTF setting for TMR and BiFC, respectively. Acquisition was performed with a pixel dwell time of 1.92 μs. Fluorescence was filtered by a band pass of 495–559 nm for BiFC and 573–627 nm for TMR. Processing was performed with the ZEN Airyscan processing module.

Super-resolution by polarization demodulation microscopy

Principles of the experimental setup are described in Hafi et al. (2014), and modifications to enable analysis of the dye's 3D orientations are described in Albrecht et al. (2020). The coverslip was fixed to the microscope slide with nail polish. Linearly polarized light derived from a 488 nm continuous-wave laser (sapphire 488-50, Coherent) was used for the excitation of Oregon Green molecules. The beam was expanded through a telescope system. The polarization was modulated at 15 frames per modulation period by rotation of a λ/2 waveplate. The rotation was achieved through a chopper wheel (Optical Chopper Systems, Thorlabs), which was synchronized to an electron-multiplying charge-coupled device (EMCCD) camera (iXon^{EM}+897 back illuminated, Andor Technology). The rotation of two wedge prisms caused lateral shift of the beam, which enabled measurements of the fluorophores being excited from a different direction. The beam was then focused onto the back aperture of the microscope objective (UPlanSAPO, 60×/1.35 NA oil immersion, Olympus), which was integrated in an inverted microscope body (IX 71, Olympus). Emitted light was then passed through a dichroic mirror (beam splitter *Z* 488 RDC, AHF) and an emission filter (ET band pass 525/50, AHF). To further magnify the image and focus it, the EMCCD camera and additional lens system were used. During the measurement, 2000 frames were recorded at approximately 32 ms per frame. The first 200 frames were discarded for calibration purposes. The raw fluorescence intensity of all modulation periods of the last 1800 frames of a measurement was used for analyses. Images were "deblurred" using deconvolution algorithms. The "blurring" function or point-spread function (PSF) was approximated using the PSF Generator plug-in for ImageJ (http://bigwww.epfl.ch/algorithms/psfgenerator/). Using the PSF, the modulating fluorescence intensities were deblurred using an iterative least-squares deconvolution while accounting for the polarization modulation. The least-squares function was minimized using the FISTA algorithm (Beck and Teboulle, 2009).

SUPPLEMENTAL INFORMATION

Supplemental information is available at *Plant Communications Online*.

FUNDING

This work was financially supported by the Deutsche Forschungsgemeinschaft (grant GRK2223/1) to R.H. and R.R.M.

AUTHOR CONTRIBUTIONS

R.M.M., H.B., P.J.W., R.R.M., R.H., and D.K. planned and designed the project. R.M.M., J.N.W., M.B., C.T., S.F., J.S., and D.K. cloned the vectors and performed plant transformation and the cLSM experiments. A.A. and R.M. handled SPoD microscopy. All authors analyzed and discussed the results. R.M.M., J.N.W., R.H., and D.K. were primarily involved in drafting the manuscript, and R.M.M. and D.K. produced figures and tables. J.S., H.B., P.J.W., and R.R.M. critically read the manuscript and improved the text; all authors finalized it. R.H. and D.K. coordinated the work.

ACKNOWLEDGMENT

We are grateful to Dr. Christin-Kirsty Baillie for scientific support and critical reading. We thank Tanja Linke for excellent technical work in our lab. Our thanks also go to Dr. Steffen Burgold, Dr. Abel Pereira da Graca, and Dr. Klaus Weisshart from Carl Zeiss Microscopy GmbH for their help with Airyscan and SIM imaging. No conflict of interest declared.

Received: November 3, 2020
Revised: May 21, 2021
Accepted: June 10, 2021
Published: June 12, 2021

REFERENCES

Albrecht, A., Pfennig, D., Nowak, J., Matis, R., Schaks, M., Hafi, N., Rottner, K., and Walla, P.J. (2020). Amplitude analysis of polarization modulation data and 3D-polarization demodulation (3D-SPoD). bioRxiv https://doi.org/10.1101/2020.03.10.986034.

Banaz, N., Mäkelä, J., and Uphoff, S. (2018). Choosing the right label for single-molecule tracking in live bacteria: side-by-side comparison of photoactivatable fluorescent protein and Halo tag dyes. J. Phys. D Appl. Phys. **52**:064002.

Beck, A., and Teboulle, M. (2009). A fast iterative shrinkage-thresholding algorithm with application to wavelet-based image deblurring. In 2009 IEEE International Conference on Acoustics, Speech and Signal Processing (IEEE), pp. 693–696.

Ben-Nissan, G., Cui, W., Kim, D.J., Yang, Y., Yoo, B.C., and Lee, J.Y. (2008). Arabidopsis casein kinase 1-like 6 contains a microtubule-binding domain and affects the organization of cortical microtubules. Plant Physiol. **148**:1897–1907.

Bhat, R.A., Lahaye, T., and Panstruga, R. (2006). The visible touch: in planta visualization of protein-protein interactions by fluorophore-based methods. Plant Methods **2**:12.

Bode, S., Quentmeier, C.C., Liao, P.N., Barros, T., and Walla, P.J. (2008). Xanthophyll-cycle dependence of the energy transfer between carotenoid dark states and chlorophylls in NPQ mutants of living plants and in LHC II. Chem. Phys. Lett. **450**:379–385.

Camacho, R., Täuber, D., and Scheblykin, I.G. (2019). Fluorescence anisotropy reloaded—emerging polarization microscopy methods for assessing chromophores' organization and excitation energy transfer in single molecules, particles, films, and beyond. Adv. Mater. **31**:1805671.

Cremer, C., and Masters, B.R. (2013). Resolution enhancement techniques in microscopy. Eur. Phys. J. H **38**:281–344.

Day, R.N., and Davidson, M.W. (2012). Fluorescent proteins for FRET microscopy: monitoring protein interactions in living cells. Bioessays **34**:341–350.

England, C.G., Luo, H., and Cai, W. (2015). HaloTag technology: a versatile platform for biomedical applications. Bioconjug. Chem. **26**:975–986.

Gehl, C., Waadt, R., Kudla, J., Mendel, R.R., and Hänsch, R. (2009). New GATEWAY vectors for high throughput analyses of protein–protein interactions by bimolecular fluorescence complementation. Mol. Plant **2**:1051–1058.

Gehl, C., Kaufholdt, D., Hamisch, D., Bikker, R., Kudla, J., Mendel, R.R., and Hänsch, R. (2011). Quantitative analysis of dynamic protein–protein interactions in planta by a floated-leaf luciferase complementation imaging (FLuCI) assay using binary Gateway vectors. Plant J. **67**:542–553.

Godin, A.G., Lounis, B., and Cognet, L. (2014). Super-resolution microscopy approaches for live cell imaging. Biophys. J. **107**:1777–1784.

Grimm, J.B., English, B.P., Chen, J., Slaughter, J.P., Zhang, Z., Revyakin, A., Patel, R., Macklin, J.J., Normanno, D., Singer, R.H., et al. (2015). A general method to improve fluorophores for live-cell and single-molecule microscopy. Nat. Methods **12**:244.

Hafi, N., Grunwald, M., Van Den Heuvel, L.S., Aspelmeier, T., Chen, J.H., Zagrebelsky, M., Schütte, O.M., Steinem, C., Korte, M., Munk, A., et al. (2014). Fluorescence nanoscopy by polarization modulation and polarization angle narrowing. Nat. Methods **11**:579.

Hamada, T. (2007). Microtubule-associated proteins in higher plants. J. Plant Res. **120**:79–98.

Holleboom, C.P., and Walla, P.J. (2014). The back and forth of energy transfer between carotenoids and chlorophylls and its role in the regulation of light harvesting. Photosynthesis Res. **119**:215–221.

Huang, B., Bates, M., and Zhuang, X. (2009). Super-resolution fluorescence microscopy. Annu. Rev. Biochem. **78**:993–1016.

Ishikawa, H., Meng, F., Kondo, N., Iwamoto, A., and Matsuda, Z. (2012). Generation of a dualfunctional split-reporter protein for monitoring membrane fusion using self-associating split GFP. Protein Eng. Des. Select. **25**:813–820.

Karimi, M., Inzé, D., and Depicker, A. (2002). GATEWAY™ vectors for Agrobacterium-mediated plant transformation. Trends Plant Sci. **7**:193–195.

Kaufholdt, D., Gehl, C., Geisler, M., Jeske, O., Voedisch, S., Ratke, C., Bollhöner, B., Mendel, R.R., and Hänsch, R. (2013). Visualization and quantification of protein interactions in the biosynthetic pathway of molybdenum cofactor in *Arabidopsis thaliana*. J. Exp. Bot. **64**:2005–2016.

Kaufholdt, D., Baillie, C.K., Bikker, R., Burkart, V., Dudek, C.A., von Pein, L., Rothkegel, M., Mendel, R.R., and Hänsch, R. (2016a). The molybdenum cofactor biosynthesis complex interacts with actin filaments via molybdenum insertase Cnx1 as anchor protein in Arabidopsis thaliana. Plant Sci. **244**:8–18.

Kaufholdt, D., Baillie, C.K., Meyer, M.H., Schwich, O.D., Timmerer, U.L., Tobias, L., van Thiel, D., Hänsch, R., and Mendel, R.R. (2016b). Identification of a protein-protein interaction network downstream of molybdenum cofactor biosynthesis in Arabidopsis thaliana. J. Plant Physiol. **207**:42–50.

Kaufholdt, D., Baillie, C.K., Meinen, R., Mendel, R.R., and Hänsch, R. (2017). The molybdenum cofactor biosynthesis network: in vivo protein-protein interactions of an actin associated multi-protein complex. Front. Plant Sci. **8**:1946.

Kerppola, T.K. (2013). Bimolecular fluorescence complementation (BiFC) analysis of protein interactions in live cells. Cold Spring Harbor. Protoc. **2013**: pdb-prot076497.

Kubitscheck, U., Kückmann, O., Kues, T., and Peters, R. (2000). Imaging and tracking of single GFP molecules in solution. Biophys. J. **78**:2170–2179.

Kudla, J., and Bock, R. (2016). Lighting the way to protein-protein interactions: recommendations on best practices for bimolecular fluorescence complementation analyses. Plant Cell **28**:1002–1008.

Lang, C., Schulze, J., Mendel, R.R., and Hänsch, R. (2006). HaloTag™: a new versatile reporter gene system in plant cells. J. Exp. Bot. **57**:2985–2992.

Lampugnani, E.R., Wink, R.H., Persson, S., and Somssich, M. (2018). The toolbox to study protein–protein interactions in plants. Crit. Rev. Plant Sci. **37**:308–334.

Lee, H.L.D., Lord, S.J., Iwanaga, S., Zhan, K., Xie, H., Williams, J.C., Wang, H., Bowman, G.R., Goley, E.D., Shapiro, L., et al. (2010). Superresolution imaging of targeted proteins in fixed and living cells using photoactivatable organic fluorophores. J. Am. Chem. Soc. **132**:15099–15101.

Liao, P.N., Bode, S., Wilk, L., Hafi, N., and Walla, P.J. (2010). Correlation of electronic carotenoid–chlorophyll interactions and fluorescence quenching with the aggregation of native LHC II and chlorophyll deficient mutants. Chem. Phys. **373**:50–55.

Liao, P.N., Pillai, S., Gust, D., Moore, T.A., Moore, A.L., and Walla, P.J. (2011). Two-photon study on the electronic interactions between the first excited singlet states in Carotenoid– tetrapyrrole dyads. The J. Phys. Chem. A **115**:4082–4091.

Liss, V., Barlag, B., Nietschke, M., and Hensel, M. (2015). Self-labelling enzymes as universal tags for fluorescence microscopy, super-resolution microscopy and electron microscopy. Sci. Rep. **5**:17740.

Loison, O., Weitkunat, M., Kaya-Çopur, A., Alves, C.N., Matzat, T., Spletter, M.L., Luschnig, S., Brasselet, S., Lenne, P.F., and Schnorrer, F. (2018). Polarization-resolved microscopy reveals a

muscle myosin motor-independent mechanism of molecular actin ordering during sarcomere maturation. PLoS Biol. **16**:e2004718.

Long, Y., Stahl, Y., Weidtkamp-Peters, S., Postma, M., Zhou, W., Goedhart, J., Sánchez-Pérez, M.-I., Gadella, T.W.J., Jr., Simon, R., Scheres, B., et al. (2017). In vivo FRET–FLIM reveals cell-type-specific protein interactions in Arabidopsis roots. Nature **548**:97–102.

Los, G., and Wood, K. (2007). The HaloTag: a novel technology for cell imaging and protein analysis. Methods Mol. Biol. **356**:195–208.

Los, G.V., Encell, L.P., McDougall, M.G., Hartzell, D.D., Karassina, N., Zimprich, C., Wood, M.G., Learish, R., Ohana, R.F., Urh, M., et al. (2008). HaloTag: a novel protein labeling technology for cell imaging and protein analysis. ACS Chem. Biol. **3**:373–382.

Mao, G., Buschmann, H., Doonan, J.H., and Lloyd, C.W. (2006). The role of MAP65-1 in microtubule bundling during Zinnia tracheary element formation. J. Cell Sci. **119**:753–758.

Miyawaki, A., Sawano, A., and Kogure, T. (2003). Lighting up cells: labelling proteins with fluorophores. Nat. Cell Biol. **Suppl**:S1–S7.

Moerner, W.E., and Kador, L. (1989). Optical detection and spectroscopy of single molecules in a solid. Phys. Rev. Lett. **62**:2535.

Morisaki, T., and McNally, J.G. (2014). Photoswitching-free FRAP analysis with a geneticall encoded fluorescent tag. PLoS One **9**:e107730.

Ohana, R.F., Encell, L.P., Zhao, K., Simpson, D., Slater, M.R., Urh, M., and Wood, K.V. (2009). HaloTag7: a genetically engineered tag that enhances bacterial expression of soluble proteins and improves protein purification. Protein Expr. Purif. **68**:110–120.

Orrit, M., and Bernard, J. (1990). Single pentacene molecules detected by fluorescence excitation in a p-terphenyl crystal. Phys. Rev. Lett. **65**:2716.

Promega. (2018). Technical Manual, HaloTag® Technology: Focus on Imaging (Madison, WI: Promega), p. 12.

Reck-Peterson, S.L., Yildiz, A., Carter, A.P., Gennerich, A., Zhang, N., and Vale, R.D. (2006). Single-molecule analysis of dynein processivity and stepping behavior. Cell **126**:335–348.

Riedl, J., Crevenna, A.H., Kessenbrock, K., Yu, J.H., Neukirchen, D., Bista, M., Bradke, F., Jenne, D., Holak, T.A., Werb, Z., et al. (2008). Lifeact: a versatile marker to visualize F-actin. Nat. Methods **5**:605.

Schindelin, J., Arganda-Carreras, I., Frise, E., Kaynig, V., Longair, M., Pietzsch, T., Preibisch, S., Rueden, C., Saalfeld, S., Schmid, B., et al. (2012). Fiji: an open-source platform for biological-image analysis. Nat. Methods **9**:676–682.

Sheahan, M.B., Staiger, C.J., Rose, R.J., and McCurdy, D.W. (2004). A green fluorescent protein fusion to actin-binding domain 2 of Arabidopsis fimbrin highlights new features of a dynamic actin cytoskeleton in live plant cells. Plant Physiol. **136**:3968–3978.

Struk, S., Jacobs, A., Sánchez Martín-Fontecha, E., Gevaert, K., Cubas, P., and Goormachtig, S. (2019). Exploring the protein–protein interaction landscape in plants. Plant Cell Environ. **42**:387–409.

Voinnet, O., Rivas, S., Mestre, P., and Baulcombe, D. (2003). An enhanced transient expression system in plants based on suppression of gene silencing by the p19 protein of tomato bushy stunt virus. Plant J. **33**:949–956.

Wallrabe, H., and Periasamy, A. (2005). Imaging protein molecules using FRET and FLIM microscopy. Curr. Opin. Biotechnol. **16**:19–27.

Xie, W., Nielsen, M.E., Pedersen, C., and Thordal-Christensen, H. (2017). A split-GFP gateway cloning system for topology analyses of membrane proteins in plants. PLoS One **12**:e0170118.

Zitnik, M., Feldman, M.W., and Leskovec, J. (2019). Evolution of resilience in protein interactomes across the tree of life. Proc. Natl. Acad. Sci. U S A **116**:4426–4433.

Plant Communications, Volume *2*

Supplemental information

Split-HaloTag imaging assay for sophisticated microscopy of protein–protein interactions *in planta*

Rieke Minner-Meinen, Jan-Niklas Weber, Andreas Albrecht, Rainer Matis, Maria Behnecke, Cindy Tietge, Stefan Frank, Jutta Schulze, Henrik Buschmann, Peter Jomo Walla, Ralf-R. Mendel, Robert Hänsch, and David Kaufholdt

Supplemental Information

Split-HaloTag® Imaging Assay for Sophisticated Microscopy of Protein-Protein Interactions *in planta*

Plant Communications

Rieke Minner-Meinen[1], Jan-Niklas Weber[1], Andreas Albrecht[2], Rainer Matis[2], Maria Behnecke[1], Cindy Tietge[1], Stefan Frank[1], Jutta Schulze[1], Henrik Buschmann[3], Peter Jomo Walla[2], Ralf-R. Mendel[1], Robert Hänsch[1,4]* and David Kaufholdt[1]

[1]Institut für Pflanzenbiologie, Technische Universität Braunschweig, Humboldtstrasse 1, D-38106 Braunschweig, Germany

[2]Institut für Physikalische und Theoretische Chemie, Technische Universität Braunschweig, Hagenring 30.023c, D-38106 Braunschweig, Germany

[3]Botany Department, Universität Osnabrück, Barbara Str. 11, 49076 Osnabrück Germany

[4]Center of Molecular Ecophysiology (CMEP), College of Resources and Environment, Southwest University, Tiansheng Road No. 2, 400715 Chongqing, Beibei District, P.R. China

*author for correspondence: Tel: +49-(0)531-391-5867
Fax: +49-(0)531-391-8128
E-Mail: r.haensch@tu-bs.de

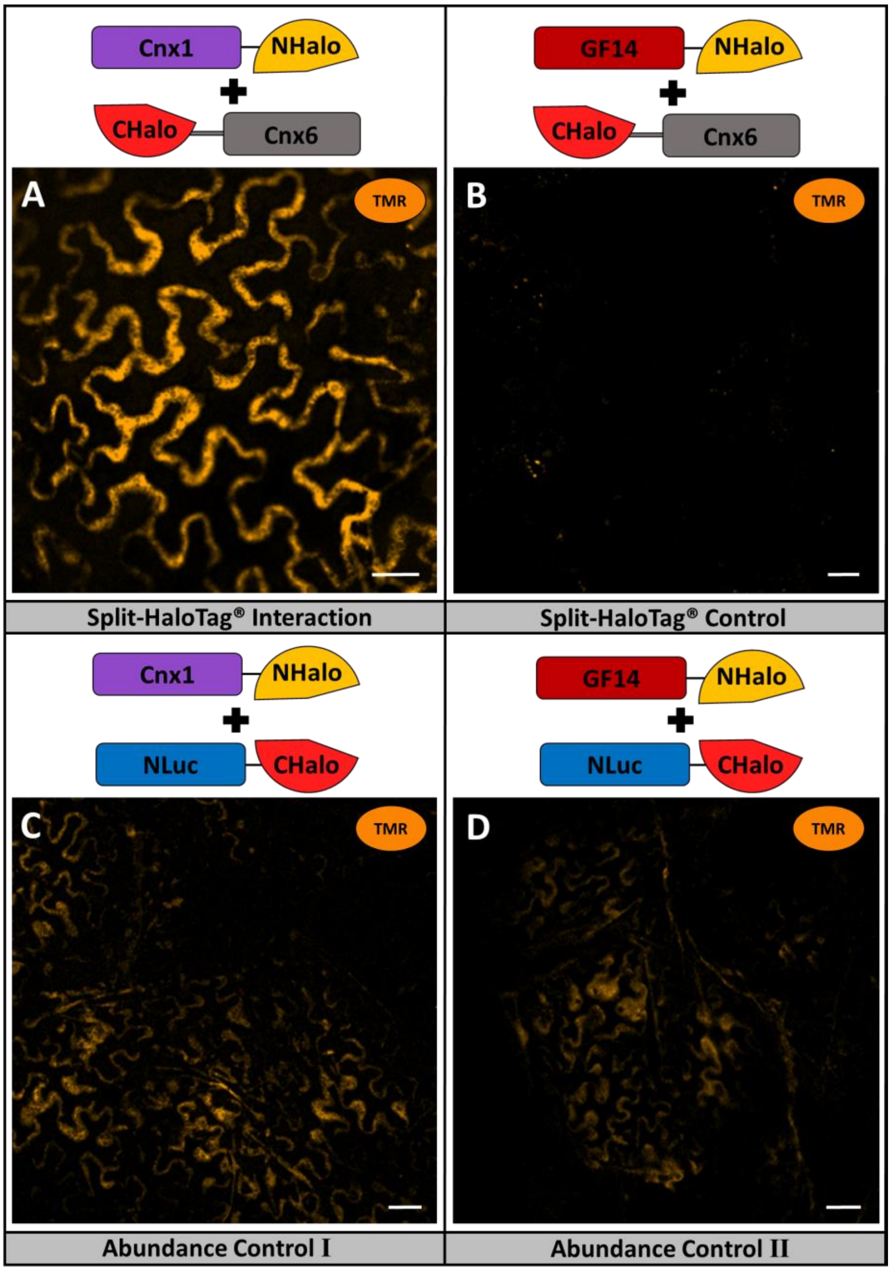

Fig. S1: Split-HaloTag® Protein-Protein Interaction Studies of Cnx1 and Cnx6 analogue to the BiFC study in Kaufholdt *et al*. (2013).

Shown are images of *N. benthamiana* epidermis cells via confocal microscopy of TMR. Staining of leaf discs was performed 5 days after transformation. All images were taken with identical setting (Plan-Neofluar 10x/0.3) for optimal comparison of fluorescence strength. Scale bars depict a length of 50 µm. **(A)** Interaction approach with Cnx1-NHalo and CHalo-Cnx6. **(B)** Negative control with the non-interacting proteins GF14-NHalo and CHalo-Cnx6. **(C/D)** Abundance control to validate negative control with **(C)** Cnx1-NHalo and NLuc-CHalo as well as **(D)** GF14-NHalo and NLuc-CHalo.

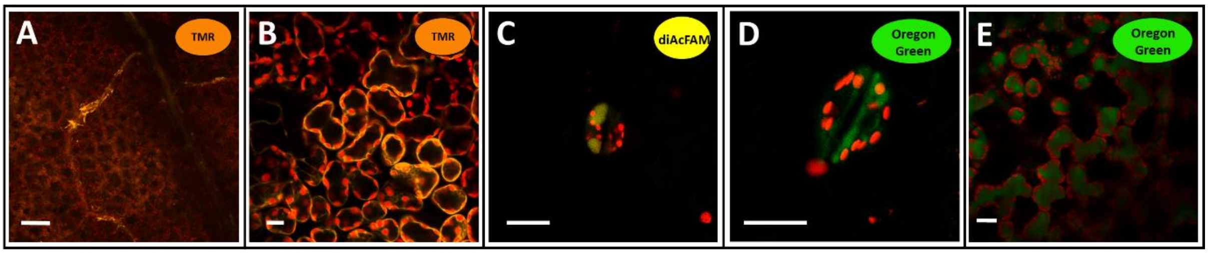

Fig. S2: Evaluation of HaloTag® fluorescent Ligands TMR, DiAcFAM and Oregon Green.
Confocal microscopy of *N. benthamiana* leaf discs stained with TMR **(A/B)**, DiAcFAM **(C)** or Oregon Green **(D/E)**. Images were taken either with a Plan-Neofluar 10x/0.3 **(A)** or with a C-Apochromat 40x/1.2 water immersion objective **(B-E)**. Scale bars depict a length of 100 µm **(A)** or 20 µm **(B-E)**. **(A)** Unspecific TMR binding in vascular tissue. **(B)** Oversaturation of unbound TMR in parenchyma cells after using 2 µM dye. **(C/D)** Accumulation of unbound ligands in stomata after DiAcFAM **(C)** and Oregon Green **(D)** staining. **(E)** Accumulation of unbound Oregon Green in parenchyma cells.

Table S1: Primers for Cloning and sequencing of Split-HaloTag® related constructs and vectors. (**a**) Cloning of Split-HaloTag® Destination vectors (restriction + ligation); (**b**) Cloning of Split-HaloTag® Fusion Constructs. All oligonucleotides are purchased from Sigma-Aldrich (Steinheim, Germany).

Name	Sequence (5´-3´)	Purpose
**XbaI_ATG_Nhalo_for**	GGGGGGTCTAGAATGGGATCCGAAATCGGTACTG	a
**NHalo_ Linker_SpeI_rev**	CAAAAACTAGTGCCGCTGCCGCCGGTGCGGAAGGCCTGGAA GGTC	a
**XbaI_ATG_Chalo_for**	GGGGGGTCTAGAATGACCGACGTCGGCCGCAAGCTGAT	a
**CHalo_Linker_ SpeI_rev**	CCAAAAACTAGTGCCGCTGCCGCCACCGGAAATCTCCAGAGT AGAC	a
**XhoI_Linker_NHalo_f or**	GGGGGGCTCGAGGGCAGCGGCGGCATGGGATCCGAAATCG GTACTG	a
**NHalo_N_SacI_rev**	CCAAAAGAGCTCTTAGGTGCGGAAGGCCTGGAAGGTC	a
**XhoI_Linker_Chalo_ for**	GGGGGGCTCGAGGGCAGCGGCGGCACCGACGTCGGCCGCA AGCTG	a
**CHalo-N_SacI_rev**	CCAAAAGAGCTCTTAACCGGAAATCTCCAGAGTAGACAGCC	a
**AttB1_N-Halo_for**	GGGGACAAGTTTGTACAAAAAAGCAGGTTTAACCATTGGATCC GAAATCGGTACTGG	b (*Nhalo-cnx7)*
**Cnx7Ueb_Linker_ N-Halo-rev**	CAATCTTTGTAACTTCTTTGTCCATGCCGCCGCTGCCGGTGC GGAAGGCCTGGAAGGT	b (*Nhalo-cnx7)*
**N- Halo_Linker_Cnx7Ue b_ for**	ACCTTCCAGGCCTTCCGCACCGGCAGCGGCGGCATGGACAA AGAAGTTACAAAGATTG	b (*Nhalo-cnx7)*
**Cnx7_Stopp_AttB2_ rev**	GGGGACCACTTTGTACAAGAAAGCTGGGTCTCAGCCGCCGCT TATCGGAGGTAT	b (*Nhalo-cnx7)*
**AttB1_C-Halo_for**	GGGGACAAGTTTGTACAAAAAAGCAGGCTTAACCATGACCGA CGTCGGCCGCAAGCTGA	b (*Chalo-cnx6*, *Chalo-map65)*
**Cnx6Ueb_Linker_ C-Halo_rev**	GGTTCTTCTCCTCTGCAGACATGCCGCCGCTGCCACCGGAAA TGTCCAGAGTAGACAGC	b *(CHalo-cnx6)*
**C- Halo_Linker_Cnx6Ue b_ for**	GCTGTCTACTCTGGAGATTTCCGGTGGCAGCGGCGGCATGTC TGCAGAGGAGGAGGACC	b (*CHalo-cnx6)*
**Cnx6_Stopp_attB2_r ev**	GGGGACCACTTTGTACAAGAAAGCTGGGTCTCAAGAAGAAGA TTTGTTATCTCCTGTAAT	b (*CHalo-cnx6)*
**Map65- 3+4_Stopp_attB2_re v**	GGGGACCACTTTGTACAAGAAAGCTGGGTCTCATGGTGAAGC TGGAACTTGATG	b (*CHalo-map65*)
**Map65-3+4- Ueb_Link_C- Halo_rev**	CATGATTCTCTCACGAGCAGAGGCAGCGGCGGCACCGGAAA TCTCCAGAGTAGACAGC	b (*CHalo-map65*)
**C-Halo- Ueb_Link_Map65- 3+4_for**	GCTGTCTACTCTGGAGATTTCCGGTGCCGCCGCTGCCTCTGC TCGTGAGAGAATCATG	b (*CHalo-map65*)

Table S2: **Split-HaloTag® Destination and Expression vectors used within this work.**

Vector	Reporter	Fusion orientation	Selectable with
**pDest-*Nhalo-GW***	HaloTag® (N-Terminus)	N-terminal	Kanamycin/Chloramphenicol
**pDest-*Chalo-GW***	HaloTag® (C-Terminus)	N-terminal	Kanamycin/Chloramphenicol
**pDest-*GW-Nhalo***	HaloTag® (N-Terminus)	C-terminal	Kanamycin/Chloramphenicol
**pDest-*GW-Chalo***	HaloTag® (C-Terminus)	C-terminal	Kanamycin/Chloramphenicol
**pExp-*Nhalo-cnx7***	HaloTag® (N-Terminus)	N-terminal	Spectinomycin
**pExp-*Chalo- cnx6***	HaloTag® (C-Terminus)	N-terminal	Spectinomycin
**pExp-*Chalo-map65***	HaloTag® (C-Terminus)	N-terminal	Spectinomycin
**pExp-*cnx1-Nhalo***	HaloTag® (N-Terminus)	C-terminal	Kanamycin
**pExp-*la-Nhalo***	HaloTag® (N-Terminus)	C-terminal	Kanamycin
**pExp-*ckl6-Nhalo***	HaloTag® (N-Terminus)	C-terminal	Kanamycin
**pExp-*la-Chalo***	HaloTag® (C-Terminus)	C-terminal	Kanamycin
**pExp-*abd2-Chalo***	HaloTag® (C-Terminus)	C-terminal	Kanamycin
**pExp-*Nluc-Chalo***	HaloTag® (C-Terminus)	C-terminal	Kanamycin
**pExp-*cnx1-vyne***	Venus (N-Terminus)	C-terminal	Kanamycin
**pExp-*abd2-scyce***	SCFP (C-Terminus)	C-terminal	Kanamycin
**pExp-*la-scyce***	SCFP (C-Terminus)	C-terminal	Kanamycin
